# Effect of the application of humanized nursing care on the clinical outcomes of neonates with hyperbilirubinemia

**DOI:** 10.1186/s12912-025-02772-z

**Published:** 2025-02-08

**Authors:** Sahar Mahmoud Elkhedr Abdelgawad, Eman Salah Elmetwaly Abdelrahman Galalah, Heba Saied El-mahdy, Nagafa Hafez Farag Elmahdy

**Affiliations:** 1 Pediatric Nursing, Faculty of Nursing, Tanta University, Tanta University, Egypt; 2 Pediatric Medicine, Faculty of Medicine, Tanta University, Tanta University, Egypt

**Keywords:** Clinical, Humanized care, Hyperbilirubinemia, Outcomes, Neonates

## Abstract

**Background:**

Neonatal hyperbilirubinemia is a potentially fatal condition that has toxic effects on the brain and may have negative effects. Humanized care is a comprehensive approach that focuses on the nutritional, therapeutic and hygienic needs of neonates, which helps in the resolution of hyperbilirubinemia.

**Method:**

A quasi-experimental design was utilized in this study to evaluate the effect of humanized nursing care application on the clinical outcomes of neonates with hyperbilirubinemia. It was carried out at the Neonatal Intensive Care Unit at Tanta Main University Hospital. A total of 120 neonates with hyperbilirubinemia who fit the inclusion criteria were randomly assigned to both the study and control groups. The data were collected using the biosocial-demographic characteristics of the studied neonates and neonatal clinical outcome measures for humanized care.

**Results:**

On the seventh day after receiving humanized care, total bilirubin levels in the study group decreased to 4.03 ± 1.48 compared to 10.21 ± 2.08 in the control group; there was also a decline in the time of jaundice regression with a mean of 3.966 ± 1.09 in the study group compared to 4.66 ± 1.29. in the control group. The mean days of phototherapy were 1.83 ± 0.73 in the study group and 2.41 ± 1.01 in the control group. Additionally, oxygen saturation increased significantly on the 7th day in the study group compared to the control group; the mean amount of daily milk intake was 752.8 ± 262.9 ml in the study group compared with 600.76 ± 290.31 ml in the control group.

**Conclusion:**

Applying humanized care improved neonatal clinical outcomes, including O_2_ saturation, jaundice regression, enhanced newborn feeding and decreased duration of phototherapy.

## Introduction

Neonatal hyperbilirubinemia is the most common reason for hospitalization in the first week of life worldwide. It may have an effect on 60% and 80% of term and preterm neonates, respectively [1, 2]. Neonatal jaundice is characterized by yellowish staining of a newborn's skin and other membranes, which indicates elevated levels of unconjugated bilirubin in the blood [1]. When a newborn's total blood bilirubin level exceeds 5 mg/dl, jaundice becomes clinically evident. Newborns may experience both pathological and physiological types of jaundice. The main causes of pathological jaundice are abnormalities in bile acid production, liver metabolism, and excessive bilirubin secretion [3]. Physiological jaundice usually disappears on its own, while pathologic jaundice frequently needs therapeutic intervention [4].

Excessive amounts of bilirubin accumulate in the bloodstream and affect the brain, possibly leading to mental retardation, hearing loss, vision impairments, or even death. When jaundice is not treated, kernicterus starts to develop, which can lead to serious problems, such as permanent brain impairment. Therefore, early diagnosis and prompt treatment to reduce bilirubin levels are essential for prevention [5].

Currently, there are numerous methods for treating hyperbilirubinemia, including medication, phototherapy, and blood exchange transfusion. However, phototherapy is used as the primary mode of treatment [6, 7]. Phototherapy involves the application of fluorescent light to newborn naked skin to facilitate bilirubin excretion through photoisomerization, which converts bilirubin's chemical structure from one that is insoluble to one that is soluble for easy excretion [8].

The environment inside neonatal intensive care units (NICUs) is characterized by high stress due to different factors, such as mechanical noise from equipment, movement of people, parallel and loud conversations, and handling of neonates. Therefore, these NICUs have been seeking ways to improve their environment, creating a more humanized environment and reducing stressful factors [9].

Humanization practices have increasingly stood out in the healthcare field, especially when present in situations in which the environment is a stressful factor for the neonate, directly impacting his/her treatment. These humanization practices include nonpharmacological treatments and noninvasive procedures that can alleviate pain, anxiety, or even fear, increase well-being and even improve neonatal clinical outcomes [10, 11].

The World Health Organization (WHO) describes "humanized care" as the act of human interaction and cooperation. It makes an effort to enhance and appreciate the essential spirit of life [12]. It has significant potential for physical and emotional intimacy between neonates and professionals, as well as between neonates and their families, from the perspective of health care intervention [13, 14]. Feeding techniques, gentle stroking, kinesthetic stimulation, and bird nest care are the major components of humanized nursing care [15, 16].

Feeding not only provides a proper supply of essential nutrients but also promotes the recovery of gastrointestinal function and a normal frequency of defecation for neonatal jaundice patients. Feeding strategies include breast feeding or artificial feeding, such as formula milk (oral or nasogastric tube) and parenteral nutrition. Adequate breastfeeding promotes normal intestinal flora establishment in newborns as well as physical growth and development, all of which help fight against physiological jaundice [17].

Consequently, most of the care given in NICUs is provided by neonatal nurses, who have the ability to modify feeding schedules to enhance feeding tolerance, assist mothers in starting and maintaining a milk supply, and facilitate the expression, handling, and storage of milk. To support the newborn's normal, healthy physical, psychological, and emotional development, nurses can also learn strategies and techniques for feeding the neonates, providing oral care and skin-to-skin care (also known as Kangaroo care), and creating opportunities for fostering parent–child interactions [18].

Additionally, nurses attach the nasogastric (NG) feeding tubes to neonates who are unable to receive oral feeding, maintain feeding tubes, provide neonates with appropriate nutrients, and avoid complications. The newborns were encouraged to sleep on their right side to reduce the possibility that they would spit up after eating. Finally, the type, amount of milk, residual volume, and any complications were recorded [19].

Neonatal massage and kinesthetic stimulation are recognized as noninvasive, supplemental treatments that do not need specialized equipment and are safe for neonates older than 31 weeks. Daily massage can enhance physiological development, improve a infant's respiration and heart rate, and improve communication between nurses and neonates. It can also alleviate colic and abdominal bloating. Moreover, massage can increase meconium elimination and prevent bilirubin from re-entering the circulatory system through the portal system, which lowers blood bilirubin levels [20, 21]. Hence, pediatric nurses should frequently stroke the whole body of neonates from top to bottom after disinfecting their hands and applying kinesthetic stimuli, such as active range-of-motion movements in the lower and upper extremities that involve changing the newborn's position between supine and prone [22].

Nesting care is a sort of developmental care that is well liked in the NICU due to its ease of application, accessibility of materials, and absence of size restrictions. It can maintain the newborn's curved limb position, minimize unexpected movement and excessive limb extension, and improve the comfort and sleep quality of the newborn by establishing boundaries around them [23].

### Significance of the study

Despite evidence-based guidelines for monitoring and treating hyperbilirubinemia, failure to prevent and manage neonatal hyperbilirubinemia results in permanent disability and even death. The humanization of care is a broad and inclusive program that includes and values excellence in the quality of care. Since it can prevent the onset of certain sequalae that affect newborns. Jean Watson supports the idea that, nurses in all settings, especially critical care units, should strengthen their humanistic role throughout the hospitalization process. Humanized care should not simply remain a theoretical concept, but rather be implemented through the recognition of the importance of the human touch and the individuality that are essential in NICUs [24–26]. However, studies of the combination between feeding strategies, gent stroking and kinesthetic stimulation and bird’s nest care as a humanized nursing care for neonates with hyperbilirubinemia are limited. Hence, this study was designed to evaluate the effect of the application of humanized nursing care on the clinical outcomes of neonates with hyperbilirubinemia.

### Aim of the study

This study was conducted to evaluate the effect of the application of humanized nursing care on the clinical outcomes of neonates with hyperbilirubinemia.

### Research hypothesis

Neonatal clinical outcomes are expected to be improved after applying humanized nursing care for neonates with hyperbilirubinemia.

### Operational definitions

1. *Humanized care* is a comprehensive strategy that promotes the discharge of newborns and attends to the nutritional, therapeutic, psychological, and hygienic aspects of neonatal nursing. In the current study, humanized nursing care included feeding strategies, gentle stroking, kinesthetic stimulation and bird’s nest care.
2. *Clinical outcomes*: is a good outcome when the neonate improved after applying humanized nursing care such as reducing bilirubin level, decrease duration of phototherapy, improve oxygen saturation, and increase amount of daily milk intake.

## Material and methods

### Design

In the present study, a quasi-experimental research design was used.

### Setting

The research was performed in the Neonatal Intensive Care Unit (NICU), Tanta Main University Hospital. Which affiliated to Ministry of Higher Education and Scientific Research in El-Gharbia Governorate, Egypt. It was conducted for a duration of one year, starting from the beginning of November 2020 to the end of November 2021. The aforementioned setting was visited four days a week, specifically on Sundays to Wednesdays, from 9.00 a.m. to 12.00 p.m. each day.

### Sample

- A sample of 120 preterm neonates was purposefully divided into two groups of 60 neonates each. The neonates were selected randomly for either the study group or the control group, based on the serial numbers of their cases. The study group received routine care as well as humanized nursing care, in contrast to the control group, while the control group received only routine care. Neonates with single numbers were selected for the study group, while those with double numbers were selected for the control group. Based on the following inclusion criteria:
- Neonates with hyperbilirubinemia, both sexes, more than 35 weeks, who were from the first day to ten days undergoing phototherapy and in the same therapeutic plan and treatment modalities. Neonates who had severe infectious disease and severe cardiac, hepatic, and renal insufficiency were excluded. The sample size was determined via power analysis, taking into account a level of significance of 95%, a study power of 80%, and a margin of error of 5% using Epi info software program.

### Two tools were used in the current study

#### Tool I: Biosocial characteristics of the studied neonates

Including gestational age, postnatal age, type of hyperbilirubinemia, weight, signs and symptoms of hyperbilirubinemia and treatment modalities.

#### Tool II: Neonatal clinical outcome: measures for humanized care

It was used to assess physiological parameters for both the study and control groups on the first and seventh days after nursing intervention. Regarding both routine care and humanized nursing care of neonates with hyperbilirubinemia. It included the following:

1. Bilirubin level: Total serum bilirubin & direct serum bilirubin.
2. Clinical efficacy: Time of jaundice regression & duration of phototherapy.
3. Using a pulse oximeter to measure oxygen saturation (the higher oxygen indicates good prognosis).
4. Milk intake: An increased milk intake indicates a good prognosis.

### Method

Two tools (I, II) were developed by the researcher after reviewing recent literature [17, 27]. To measure the study tools' use, visibility, and clarity, a pilot study was conducted on ten neonates (10% of the sample). After the necessary modifications were made, those neonates were excluded from the study's total sample.

#### Phases of the study

The study was carried out in four phases:

1. Assessment phase

Baseline data on neonates were extracted from their hospital records on the first day of admission for the two groups (control & study) before intervention to select neonates who fulfilled the inclusion criteria of the present research (Tool I). The data were initially gathered from the control group and then from the group receiving humanized care to prevent the subjects from contaminating.

2. Planning phase
  *- For the control group*, neonates only received routine care for hyperbilirubinemia, including incubator care, eye care, skin care, genitalia care, phototherapy care (covering eyes, positioning, hydration, monitoring serum bilirubin, and side effects of phototherapy) and care of the peripheral intravenous line care.
  *- For the study group*: humanized nursing care began on the first day; the neonate received phototherapy and continued through the seventh day. It included the following items: *feeding strategies*, which include the type of feeding, frequency, digestive capacity, and amount of milk. *Gentle stroking*: the researcher massaged the neonates' entire body, from head to toe. *Kinesthetic stimulation* included passive motion of the limbs singly and then together in the following order: right arm, left arm, right leg, and left leg. For the *bird’s nest care*, the bath towel was rolled and folded around the newborn to promote security for the neonate

3. Implementation phase: (For the study) group

#### During the first stage

The neonate was placed in the supine position and gently stroked in the following sequence:

#### Gentle stroking technique

Stroking was performed once in the morning, once at midnight and once in the evening from the first day to the seventh day for 15 min. Hands were carefully washed; the room temperature was maintained between 24 and 28 °C, and pressure was applied to the newborn’s skin with warm and bare hands. The periorbital and cheek regions of the face were first massaged gently with the two thumbs. It then moved to the chest by moving the two hands alternately from the lower edge of the chest to the higher edge. It then moved to the abdomen by gently pushing in a half-circle that matched the structure of the colon. After that, the outside of the upper and lower limbs was massaged with moderate pressure. Finally, the neonate was massaged from the vertebra to the sides of the neck and buttocks with both hands was performed. The massage was stopped and restarted after everything returned to normal if the newborn cried or defecated during the massage.

#### During the second stage

The neonate was positioned in the supine position, and six passive motions of the limbs were performed singly and then together. Each of the movements lasted for approximately 10 s in the following order: passive motion of the right arm with fixation along the collarbone, then the left, then the arms together; passive motion of the left arm with fixation along the collarbone, then the left, then the arms together. Passive motion of the right leg involved fixing along the pelvic bones while holding the ankle joint; passive motion of the left leg; simultaneous movements of the legs; and finally bending and straightening the two legs together. During phototherapy, the neonate was placed into the nest in the incubator (preheated to 33–34 °C) with the neck stretched and both hands and legs curved relative to the middle of the body. The bath towel was folded into an oval shape similar to a roller (a U shape).

4. Evaluation phase

Neonatal clinical outcomes, including total serum bilirubin, direct serum bilirubin, oxygen saturation, amount of daily milk intake, time of jaundice regression and duration of phototherapy, were evaluated on the 1st and 7th days after routine care and humanized nursing care (Tool II).

5. Statistical analysis

Using SPSS software, the collected data were arranged, tabulated, and statistically evaluated. The range, mean, and standard deviation were computed for quantitative data. When comparing qualitative data between two groups, among other comparisons, the chi-square test was used to compare more than two means of parametric data. A P value of 0.05 was used as the significance level for interpreting the findings of the significance tests.

## Results

### Percentage distribution of the studied neonates according to bio sociodemographic characteristics

Table 1: No statistically significant differences were observed between the study and control groups for gestational age, current age, feeding type and current weight.

**Table 1:** Percentage distribution of the studied neonates according to bio sociodemographic characteristics

**Bio sociodemographic data**	**Study group (*n* = 60)**		**Control group (*n* = 60)**		**χ2**	**P**
	**No**	**%**	**No**	**%**		
**Gestational age (weeks)**						
35 – 36	22	36.7	28	46.7	0.617	0.432
37 and more	38	63.3	32	53.3		
**Mean ± SD**	**37.43 ± 1.794**		**36.83 ± 1.510**		**t test = **1.401 *P* = 0.166	
**Current age**						
1 – 7 days	50	83.3	24	73.3	0.884	0.347
8 – 15 days	10	16.7	16	26.7		
**Type of feeding**						
Bottle feeding	32	53.3	30	50.0	0.067	0.796
Gavage feeding	28	46.7	30	50.0		
**Current weight**						
Less than 2.500 gm	20	33.3	20	33.3	0.000	1.000
2.500—4,000 gm	40	66.7	40	66.7		
**Mean ± SD**	**2.680 ± 0.368**		**2.540 ± 0.561**		**t test = **1.142	
					*P* = 0.259	

### Percentage distribution of neonatal clinical outcome measures regarding bilirubin levels on the first and seventh days after intervention

As shown in Table 2, highly statistically significant differences were found between the study and control groups on the seventh day regarding total serum bilirubin (P_2_ = 0.0001) and direct serum bilirubin (P_2_ = 0.0001), despite the nonsignificant difference between the two groups on the 1st day.

**Table 2:** Percentage distribution of neonatal clinical outcome measures regarding bilirubin levels on the first and seventh days after intervention

**Bilirubin level**	**First day (*n* = 60)**				**P1**	**Seventh day (*n* = 60)**				**P2**
	**Study group (*n* = 30)**		**Control group (*n* = 30)**			**Study group (*n* = 30)**		**Control group (*n* = 30)**		
	**No**	**%**	**No**	**%**		**No**	**%**	**No**	**%**	
**Total serum bilirubin**										
< 7 mg/dl	4	6.7	0	0.0	2.069	58	96.7	8	13.3	**42.088**
≥ 7 mg/dl	56	93.3	60	100.0	0.150	2	3.3	52	87.7	0.0001**
					1.705					**13.209**
**Mean ± SD**	**9.20 ± 2.08**		**10.01 ± 1.58**		**P 1 = **0.093	**4.03 ± 1.48**		**10.21 ± 2.08**		P2 = 0.0001**
**Direct serum bilirubin**										
< 1 mg/dl	28	46.7	22	36.7	0.617	60	100.0	24	40.0	**25.714** 0.0001**
≥ 1 mg/dl	32	53.3	38	63.3	0.432	0	0.0	36	60.0	
			**0.5 – 2.3**		1.204					**9.121** P2 = 0.0001**
**Mean ± SD**	**0.94 ± 0.29**		**1.04 ± 0.38**		**P1 = **0.233	**0.35 ± 0.12**		**1.01 ± 0.45**		

^*^Difference that is statistically significant at *P* < 0.05

^**^Statistically significantly different at (*P* < .0001), highly significant

### Percentage distribution of neonatal clinical outcomes regarding jaundice regression and duration of phototherapy after intervention

Table 3: It was obvious that jaundice regression was better in the study group than in the control group, with a mean of (3.966 ± 1.09) (4.66 ± 1.29) days, respectively, with a statistically significant difference (*P* = 0.028) between the two groups. Additionally, the duration of phototherapy was significantly shorter in the study group (mean = 1.83 ± 0.73 days), than in the control group (2.41 ± 1.01; *P* = 0.014).

**Table 3:** Percentage distribution of neonatal clinical outcomes regarding jaundice regression and duration of phototherapy after intervention

**Variables**	**Study group (*n* = 60)**		**Control group (*n* = 60)**		**χ2**	**P**
	**No**	**%**	**No**	**%**		
**Time of jaundice regression (Day)**						
≤ 3	24	23.3	10	16.7	**4.022**	0.045*
> 3 – 6	36	76.7	50	83.3		
**Mean ± SD**	**3.966 ± 1.09**		**4.66 ± 1.29**		**t test = **	0.028*
					**2.258**	
**Duration of phototherapy (Day)**						
≤ 2	46	76.7	34	56.7	**2.700**	0.001**
> 2 – 4	14	23.3	26	43.3		
**Mean ± SD**	**1.83 ± 0.73**		**2.41 ± 1.01**		**t test = 2.545**	0.014*

^*^ Difference that is statistically significant at *P* < 0.05

^**^Statistically significantly different at (*P* < .0001), highly significant

### Percentage distribution of neonatal clinical outcomes regarding oxygen saturation and amount of daily milk intake on the first and seventh days after intervention

As presented in Table 4, it was shown that oxygen saturation increased significantly on the 7th day in the study group compared to the control group, with statistically significant differences (*P* < 5.455 and *P* < 0.020) in terms of percentage and mean, respectively. Additionally, the amount of daily milk intake increased on the 7th day of intervention when compared to that in the study group and the control group, with a mean (752.8 ± 262.9 ml) (600.76 ± 290.31 ml), respectively, with a statistically significant difference between the two groups (*P* = 0.026).

**Table 4:** Percentage distribution of neonatal clinical outcomes regarding oxygen saturation and amount of daily milk intake on the first and seventh days after intervention

**Variables**	**First day (*n* = 60)**				**P 1**	**Seventh day (*n* = 60)**				**P 2**
	**Study group (*n* = 60)**		**Control group (*n* = 60)**			**Study group (*n* = 60)**		**Control group (*n* = 60)**		
	**No**	**%**	**No**	**%**		**No**	**%**	**No**	**%**	
**Oxygen saturation**										
92- 100%	50	83.3	46	76.7	0.417	60	100.0	50	83.3	**5.455**
Less than 92%	10	16.7	14	23.3	0.519	0	0.0	10	16.7	0.020*
**Amount of daily milk intake (ml/kg)**										
< 500	22	36.7	28	46.7	0.617 0.432	10	16.7	22	36.7	**7.276** 0.026*
500— < 1000	38	63.3	32	53.3		40	66.6	38	63.3	
≥ 1000	0	0.0	0	0.0		10	16.7	0	0.0	
**Mean ± SD**	**485.7 ± 267.4**		**456.4 ± 246.1**		**t test** 0.440 P1 = 0.660	**752.8 ± 262.9**		**600.76 ± 290.31**		**t test** **2.285** P2 = 0.026*

^*^Difference that is statistically significant at *P* < 0.05

^**^Statistically significantly different at (*P* < .0001), highly significant

### Correlation between neonatal biosocial characteristics and their clinical outcome measures before intervention

Table 5 No statistically significant correlations were observed between neonatal biosocial characteristics and clinical outcomes before intervention in the study or control group.

**Table 5:** Correlation between neonatal biosocial characteristics and their clinical outcome measures before intervention

**Biosocial characteristics data**	**Total serum bilirubin**		**Direct serum bilirubin**		**Oxygen saturation**		**Amount of daily milk intake (ml/kg)**	
	**r**	**P**	**R**	**P**	**R**	**P**	**r**	**P**
**Gestational age (weeks)**								
Study group	0.273	0.145	0.041	0.830	0.008	0.965	0.078	0.683
Control group	0.138	0.468	0.261	0.163	0.165	0.384	0.322	0.227
**Current age**								
Study group	0.097	0.609	0.163	0.389	0.116	0.540	0.143	0.452
Control group	0.070	0.711	0.091	0.631	0.208	0.270	0.245	0.191
**Type of feeding**								
Study group	0.078	0.682	0.074	0.696	0.188	0.319	0.065	0.731
Control group	0.206	0.274	0.089	0.640	0.050	0.795	0.101	0.594
**Current weight**								
Study group	0.165	0.383	0.117	0.537	0.065	0.733	0.079	0.679
Control group	0.002	0.991	0.105	0.580	0.237	0.207	0.184	0.330
**Treatment modalities**								
Study group	0.065	0.731	0.062	0.744	0.273	0.145	0.140	0.459
Control group	0.126	0.505	0.142	0.454	0.040	0.834	0.247	0.189

### Correlation between neonatal biosocial characteristics and their clinical outcome measures for the study and control groups after intervention

The correlations between neonatal clinical outcomes and their biosocial characteristics after intervention are illustrated in Table 6. A highly significant positive correlation was found between the type of feeding and the amount of daily milk intake in the study group (*P* = 0.004). Significant positive correlations were also found between current weight, direct serum bilirubin, oxygen saturation and amount of milk (*P* = 0.032, 0.022 & 0.049), respectively, after intervention. Additionally, a significant positive correlation between treatment modality and oxygen saturation after intervention was found in the study group (*P* = 0.046). No statistically significant correlation was found between variables in the control group.

**Table 6:** Correlation between neonatal biosocial characteristics and their clinical outcome measures for the study and control groups after intervention

**Biosocial characteristics data**	**Total serum bilirubin**		**Direct serum bilirubin**		**Oxygen saturation**		**Amount of daily milk intake (ml/kg)**	
	**R**	**P**	**R**	**P**	**r**	**P**	**r**	**P**
**Gestational age (weeks)**								
Study group	0.225	0.233	0.086	0.650	0.435	0.554	0.280	0.134
Control group	0.125	0.511	0.224	0.234	0.137	0.469	0.211	0.263
**Current age**								
Study group	0.276	0.140	0.054	0.777	0.326	0.321	0.230	0.221
Control group	0.002	0.992	0.036	0.850	0.224	0.229	0.270	0.149
**Type of feeding**								
Study group	0.149	0.431	0.012	0.951	0.712	0.621	0.514	0.004**
Control group	0.259	0.166	0.052	0.784	0.227	0.227	0.129	0.495
**Current weight**								
Study group	0.220	0.243	0.867	0.032*	0.438	0.022*	0.362	0.049*
Control group	0.037	0.846	0.081	0.672	0.150	0.428	0.107	0.575
**Treatment modalities**								
Study group	0.011	0.953	0.037	0.846	0.365	0.046*	0.148	0.437
Control group	0.040	0.834	0.139	0.464	0.932	0.932	0.218	0.248

^*^Difference that is statistically significant at *P* < 0.05

^**^Statistically significantly different at (*P* < .0001), highly significant

## Discussion

Neonatal hyperbilirubinemia is a life-threatening condition that can lead to severe jaundice and hospital readmissions. Neonatal morbidity and mortality are caused by untreated neonatal jaundice and kernicterus (Olusanya et al., 2018) [25]. The most common type of treatment for hyperbilirubinemia is phototherapy because it is effective and noninvasive. However, it is associated with severe adverse effects such as dehydration, retinal damage, and bronzy baby syndrome. As a result, it makes sense to use adjuvant interventions, such as humanized care, can improve neonatal recovery by enhancing nutritional, circulatory, mood, and vagal excitability for bilirubin elimination (Yan 2021, Ahmed Y 2019) [17, 28].

The present study was conducted to evaluate the effect of the use of humanized nursing care on the clinical outcomes of neonates with hyperbilirubinemia. According to the biosocial sociodemographic characteristics of the studied neonates, the results of the current study showed that more than half of them had gestational ages of 37 weeks or older, and more than two-thirds of them had a normal weight at the time of the study. This could be attributed to the fact that half of the neonates in the present study were born at term. These results were comparable to those of Demis et al. (2021), [29] who discovered that more than half of the neonates under study were between 37- and 42-weeks gestational age.

Furthermore, Saeedi et al. (2020) [30] found that almost the same percentage of the subjects' weight ranged from 2.500 to 4,000 kg in their study to evaluate neonatal jaundice on the first day of life, as in the present study. In addition, the results of the present study are compatible with the findings of the study conducted by Eta et al. (2023) [31], who mentioned that the majority of the neonates in their study had LBW less than 2500 g.

Additionally, the current study revealed that more than three-quarters of the participants in the study and control groups’ ages ranged from 1 to 7 days, which may be related to neonatal jaundice, which is frequent during the first week of life. These findings were consistent with those of Ahmed A et al. (2022), [32], who reported that more than two-thirds of the neonates were now between three and seven days old. Regarding the type of feeding, the current study indicated that more than half of the study and control groups received artificial feed, which was similar to the findings of Kiros et al. (2023) [33], who found that more than two-thirds of LBW infants were fed artificial milk.

Concerning treatment modalities, the current study showed that all studied neonates were receiving phototherapy. This may be justified by the importance of phototherapy as a widely used worldwide modality for treating neonatal jaundice. The findings of a study by Mukherjee et al. (2018) [34], supported this result and revealed that the majority of the neonates in the study were receiving phototherapy. Additionally, these results agreed with those of Al Gameel (2023) and Asefa et al. (2020) [35, 36], who reported that more than three-quarters and approximately two-thirds of neonates, respectively, were treated with phototherapy.

The current study demonstrated improvements in neonatal clinical outcome measures after the implementation of humanized care. There were highly significance differences in total and direct serum bilirubin between the neonates in the study and the control group. Total and direct serum bilirubin levels were significantly lower in the study group than in the control group on the seventh day.

 These results might be explained scientifically by the fact that gentle stroking and kinesthetic stimulation stimulate lymphatic blood flow and circulation, which accelerate gastrointestinal motility and result in more frequent stools with high bilirubin content. In addition, increased milk consumption results in more frequent bowel movements, which will reduce the enterohepatic circulation of bilirubin and improve bilirubin excretion [37, 38]. These findings are consistent with those of Jalalodini et al. (2016) [39], who found that tactile-kinesthetic stimulation can impact the reduction of bilirubin in neonates. Additionally, these improvements are in line with those of Wang et al. (2020) [40], who used a variety of nursing interventions to treat neonatal hemolytic disease and discovered that the mean bilirubin level in the intervention group on the seventh day of treatment was significantly lower than that in the control group.

Furthermore, the findings of the present study were also supported by the findings of Korkmaz et al. (2020) [41], who evaluated the effects of massage therapy on indirect hyperbilirubinemia in newborns receiving phototherapy and reported that the total serum bilirubin level was significantly lower in the intervention group than in the control group.

The present study reported that the time of jaundice regression in the study group was significantly shorter than that in the control group. This result was congruent with the findings of Wei et al. (2017) and Dai et al. (2021) [42, 43], as they reported a considerably short duration of jaundice regression in the study group in the neonates who received comprehensive nursing intervention for neonatal hyperbilirubinemia.

Additionally, the current study showed that the duration of phototherapy was significantly shorter in the study group than in the control group. This could be explained by the way in which humanized nursing care, such as feeding techniques, gentle touching, kinesthetic stimulation, and bird's nest care, increases the comfort and health of neonates. This shortens the course of phototherapy, shortens hospital stays, and lowers hospital expenses. Consequently, the use of humanized nursing care results in more desirable nursing impacts. This result was consistent with a study by Kenari et al. (2020) [44], who reported that the duration of phototherapy and hospital stay were much shorter in the intervention group than in the control group.

According to the current findings, on the seventh day, there was a statistically significant difference between the study and control groups in terms of oxygen saturation, but on the first day, there was no such difference.

The scientific explanation for these findings is that gentle strokes and kinesthetic stimulation increase epinephrine production, affect beta-adrenergic receptors in the airways, increase their diameter, and hence increase alveolar ventilation. Finally, this procedure increases the mean oxygen saturation. Additionally, the neonates breathed better and were calmer due to the bird’s nest technique, which increased oxygenation [45]. This result was supported by Ramezani et al. (2017) and Jazayeri et al. (2021) [46, 47], who reported that there was a significant increase in oxygen saturation after intervention compared to before intervention.

Concerning the amount of daily milk intake, the present study revealed that it was significantly higher in the study group than in the control group on the seventh day. This may be attributed to the influence of humanized care on easier environmental adaptation, which made the newborns calmer and more comfortable and increased milk intake, which improved digestive function by promoting the digestion and absorption of food and increasing body weight.

This finding was in agreement with Jiao (2023) [48], who reported that comfort care significantly increases milk intake and causes neonates to gain weight more quickly in the study group than in the control group. In addition, the study results were congruent with those of Rashwan et al. (2023) [49], who found that the intervention bundle successfully decreased the blood bilirubin level, increased the percentage of consumed milk, increased weight gain, and shortened the duration of phototherapy.

Regarding the correlation between neonatal biosocial characteristics and their clinical outcome measures after intervention, the current study showed that there were significant positive correlations between type of feeding, current weight, and amount of daily milk intake after intervention. Significant positive correlations were also found between current weight, treatment modalities, and oxygen saturation after intervention, while there was a significant negative correlation between current weight and total bilirubin level after intervention.

These findings are similar to those of Paulsamy et al. in 2021 [50], who investigated the impact of massage with or without kinesthetic stimulation on weight gain in preterm neonates and discovered that gentle stroking combined with kinesthetic stimulation had a favorable impact on weight gain and neonatal outcomes. In the study group following the intervention, there was a strong positive correlation between the amount of milk consumed daily, the type of feeding, and the current weight.

## Conclusion

Based on the findings of the present study, it can be concluded that humanized nursing care had a positive effect on improving neonatal clinical outcomes when the study group was compared to the control group by increasing the amount of daily milk intake and weight, increasing oxygen saturation, decreasing bilirubin levels, and finally decreasing the duration of phototherapy.

## Recommendations

- The application of humanized nursing care should be endorsed as a part of routine care for neonates with hyperbilirubinemia to improve their clinical outcomes.
- Educational and training programs must be provided to pediatric and neonatal nurses to apply humanized care in their practice.
- Using Humanized care for different newborns to examine its' effect and clinical outcome for other diagnosis.

## Data Availability

The datasets used and analyzed during the current study are available from the corresponding author on reasonable request.
